# Factors Associated with the Extent of Clinical Attachment Loss in Periodontitis: A Multicenter Cross-Sectional Study

**DOI:** 10.3390/ijerph20227043

**Published:** 2023-11-09

**Authors:** Gloria Inés Lafaurie, María Alejandra Sabogal, Adolfo Contreras, Diana Marcela Castillo, Diego Fernando Gualtero, Juliette De Avila, Tamy Goretty Trujillo, Andrés Duque, Astrid Giraldo, Silvia Duarte, Sonia Jakeline Gutierrez, Carlos Martín Ardila

**Affiliations:** 1 Unit of Oral Basic Investigation-UIBO, School of Dentistry, El Bosque University, Bogota 11001, Colombia or lafauriegloria@unbosque.edu.co (G.I.L.); sabogalmaria@unbosque.edu.co (M.A.S.); castillodiana@unbosque.edu.co (D.M.C.); gualterodiego@unbosque.edu.co (D.F.G.); dequiroga@unbosque.edu.co (J.D.A.); gorettytamy@unbosque.edu.co (T.G.T.); 2 Periodontal Medicine Group, Universidad del Valle, Cali 760042, Colombia; adolfo.contreras@correounivalle.edu.co (A.C.); sonia.gutierrez@correounivalle.edu.co (S.J.G.); 3 Research Group in Basic Sciences and Clinical Dentistry, CES University, Medellin 050021, Colombia; aduqued@ces.edu.co (A.D.); astridgiraldoa@gmail.com (A.G.); 4 Dental Research Center-CIO, Pontificia Universidad Javeriana, Bogota 110311, Colombia; s-duarte@javeriana.edu.co; 5 Biomedical Stomatology Research Group, Faculty of Dentistry, Universidad de Antioquia UdeA, Medellin 050010, Colombia

**Keywords:** periodontitis, IgG antibodies, *Porphyromonas gingivalis*, *Aggregatibacter actinomycetemcomitan*, smoking, plaque index

## Abstract

Periodontitis has significant public health implications, affecting individuals’ overall health, well-being, and quality of life. This study aimed to assess the risk factors associated with the extent of clinical attachment loss (CAL) in a population diagnosed with periodontitis. Six hundred and sixty-seven patients with different degrees of CAL (mild, n = 223; moderate, n = 256; and advanced, n = 188) were enrolled. Socio-demographics, lifestyle, microbiological profiles, specific immune response, obesity, and single-nucleotide polymorphism of the IL1 gene were determined. Unconditional logistic regression models were conducted to determine the factors associated with the extent of CAL. Aging, smoking, microbial factors, plaque index, and IgG2 antibodies against *Aggregatibacter actinomycetemcomitans* were associated with advanced CAL. IgG2 antibodies against *A. actinomycetemcomitans* (OR 1.50; CI 95% 1.23–1.81), plaque accumulation (OR 2.69; CI 95% 2.20–3.29), *Porphyromonas gingivalis* (OR 1.93; CI 95% 1.35–2.76), *Tanerella forsythia* (OR 1.88; CI 95%1.30–2.70), and current smoking (OR 1.94; CI 95% 1.31–2.87) were associated with advanced CAL. Gene IL polymorphisms, obesity, and stress were not associated with the extent of CAL. Aging, plaque accumulation, smoking, and having antibodies against *A. actinomycetemcomitans* were the most critical factors associated with advanced CAL. In contrast, obesity, stress, and gene polymorphisms were not associated with the extent of CAL.

## 1. Introduction

Periodontitis is a chronic inflammatory oral condition characterized by the dysbiotic alteration of the oral microbiota, resulting in an imbalance in the composition of the microbial community within the periodontal biofilm. Dysbiosis in periodontitis involves a shift from a commensal microbiota to a pathogenic one, typically marked by an overabundance of periodontal pathogens. This microbial dysbiosis triggers a host immune response, leading to sustained inflammation and the progressive destruction of the periodontal tissues, ultimately resulting in tooth-supporting structure loss and potential tooth mobility [1,2]. Periodontitis is one of the most frequent causes of tooth loss and affects large populations worldwide. This condition can have significant public health implications due to its prevalence and potential impact on overall health. By addressing risk factors and promoting preventive measures, public health initiatives can help reduce the prevalence and impact of periodontitis, leading to improved oral health and overall well-being for the population [1]. Evidence has shown an increase in the prevalence and severity of periodontitis [2]; however, the results are influenced by the level of socio-economic development [2,3,4]. Both the severity and extent of periodontal disease increase with age [5] and are influenced by tobacco smoking, specific subgingival microorganisms, and uncontrolled diabetes [6,7,8]. However, local factors such as calculus and dental plaque are also recognized in periodontitis progression [9]. Genetic aspects, in particular, the increase in the allelic frequency of single-nucleotide polymorphisms (SNPs) for IL-1 beta (IL-1b) (+3954), SNP IL alpha −889 (IL-1a), and a positive genotype of IL gene increased risk of developing periodontitis within the European Caucasian [9,10]. However, it is still controversial in Latin American populations [11,12,13]. Likewise, an association between periodontitis and putative risk factors such as high levels of stress [13,14,15] and obesity [16,17,18] has been reported.

Clinical attachment loss (CAL) is the best marker of periodontal breakdown, and this has been used to study the risk indicators for the severity of periodontal disease among the adult population [19,20]. However, a few studies evaluate the association of classic and putative factors with the extent of CAL in multivariate analyses. The hypothesis of this study posits that advanced CAL is correlated with certain established and potential risk factors.

The aim of this study was to assess the correlation between geographic, genetic, microbiological, and immunological factors, lifestyle, and obesity, and their influence on the extent of clinical attachment loss within a population through multifactorial modeling.

## 2. Materials and Methods

### 2.1. Population

**Figure 1:**
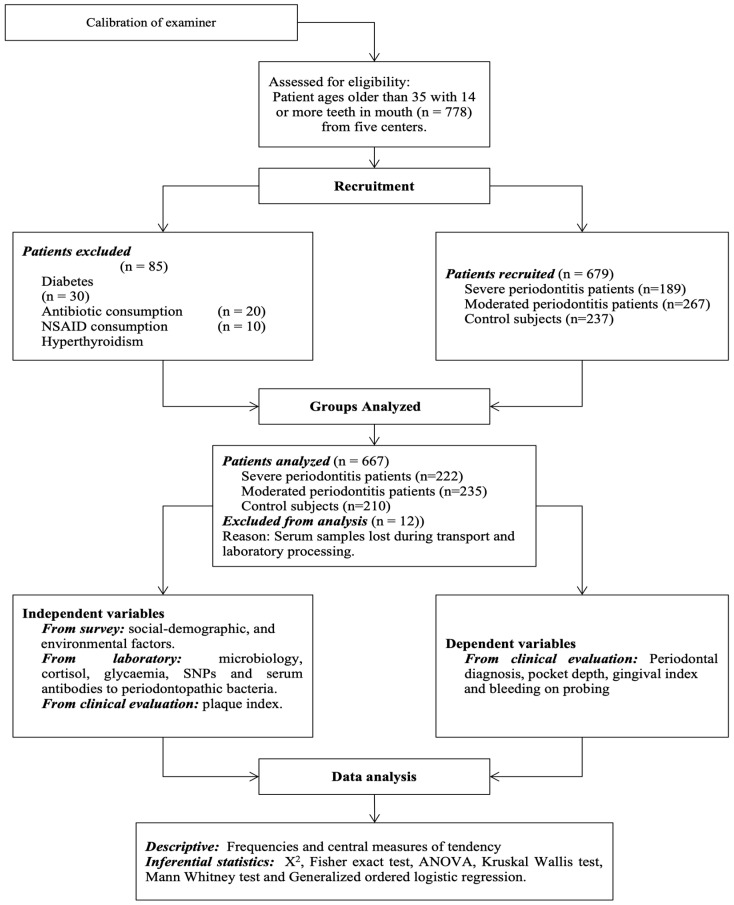
Flowchart of study protocol.

Six hundred and sixty-seven untreated adult patients over 35 years old with at least 14 teeth were studied. The patients belonged to 3 cities in Colombia: Bogotá, Medellin, and Cali, and they were attending advanced periodontal programs at 5 universities. The Institutional Review Board of each participating university approved this study, and the patients signed their informed consent. Previous periodontal treatment, antibiotic intake, immunosuppressive medication, non-steroidal anti-inflammatory drugs (NSAIDs), diagnosed diabetes, and autoimmune diseases were exclusion criteria (Figure 1).

### 2.2. Clinical Evaluation

A complete periodontal examination was performed by using the CP15 probe to measure clinical attachment loss (CAL). The included 667 individuals were categorized into three groups based on the mean of CAL of the whole dentition. (1) Mild CAL included individuals with ≤2 mm CAL on average; (2) moderate CAL included those having >2/<4 mm; and (3) advanced CAL included those having ≥4 mm [20]. The exam was carried out by three calibrated periodontists with an Intra-examiner (IE) Intra-Class Correlation Coefficient (IE- ICC) of 0.90–0.98 in each city. Other data obtained during the clinical examination were bleeding on probing (BOP) (IE-kappa index 0.75–0.95), plaque index (PI) (IE-kappa index 0.85–0.96), gingival index (GI) (IE-kappa index 0.75–0.90), and pocket depth (IE-kappa index 0.95–0.98). A middle plaque index refers to a score of 2, while a high plaque index refers to a score of 3. These scores indicate a substantial accumulation of plaque on the teeth [21].

### 2.3. Sample Size

The sample size was calculated with a power of 80%, and an error α of 5% for detecting an odds ratio (OR) ≥ 2 for risk indicators with a prevalence ≥ 15% in the control population (mild CAL). A minimum of 200 patients were calculated in each group. However, although 223 patients with mild CAL and 256 with moderate CAL were included, only 188 with advanced CAL could be enrolled.

### 2.4. Socio-Demographic Features and Lifestyle

A questionnaire was used for establishing the socio-demographics and habits including current smoking, smoking history, and alcohol intake. In addition, a blood sample was taken from every patient for carrying out a test of serum cotinine, according to the manufacturer’s guidelines to verify the tobacco habit in which a level ≥ 10 ng/mL was taken as indicative of current smoking, and serum cortisol for determining the levels of stress. Finally, the body mass index (BMI), the complete blood count (CBC), and levels of post-prandial glycemia were also assessed.

### 2.5. Single-Nucleotide Polymorphism (SNP) Analysis of IL 1 Gene by Polymerase Chain Reaction (PCR)

To determine the polymorphism of SNPs from interleukin IL-1a (-889) and interleukin IL-1b (+3954), cells were collected from the oral mucosa by mouthwash and swab sampling. To obtain DNA, the epithelial cells were subjected to extraction with the Chelex-100 resin [22]. After the sample was cooled down and the sediment allowed to settle, 40 µL supernatant was reconstituted in 10 µL of buffer Tris- Borato EDTA 5X (TBE). Samples were frozen at −20 °C until the process. Analysis of the SNP of IL-1b and IL-1a used the primers reported by Kornman et al. [23]. The reaction was amplified by initial denaturation at 94 °C for 3 min followed by 30 cycles with a temperature of 94 °C, 53 °C, and 72 °C for 1 min to IL-1b, and 94 °C, 53 °C, and 72 °C for 45 sec to IL-1a. Following amplification, the digestion of the amplification products was carried out with 4U of Taq I for 2 h at 65 °C for IL-1b and with 5U of NcoI for 16 h at 37 °C for IL-1a^††^. The fragments of enzymatic restriction were displayed in agarose gels after staining with ethidium bromide.

The following electrophoretic profile set the genotypic assay: for IL-1b, fragments of 97, 85, and 12 pb (genotype CC, absence of polymorphism), fragments of 12 and 182 pb (genotype TT, presence of polymorphism), fragments of 182, 97, 85, and 12 pb (genotype CT, presence of polymorphism on one of its alleles); for IL-1a, fragments of 83 + 16 pb (genotype CC absence of polymorphism), fragments of 99 pb (genotype TT, presence of polymorphism), fragments of 99, 83, and 16 pb (genotype CT, presence of polymorphism on one of its alleles). Analysis of Hardy–Weinberg balance on genotypic frequencies was performed by Chi-square (X^2^) test.

### 2.6. Microbiological Aspects

Supragingival plaque was eliminated using curettes, and subsequently, sterile paper points were inserted for a duration of 20 s into the six deepest periodontal sites within each subject for PCR detection of *Porphyromonas gingivalis*, *Tannerella forsythia*, *Treponema denticola*, and *Aggregatibacter actinomycetemcomitans*. *P. gingivalis* yields a band corresponding to 404 bp; *T. forsythia* yields a band of 641 bp; *T. denticola* yields a band of 316 bp; and *A. actinomycetemcomitans* yields a band of 557 bp after electrophoresis in agarose of 1.5% with 0.5 µg/mL of ethidium bromide. Reference strains of each bacterium were used as positive DNA controls. The negative control was sterile water added to the master mix [24].

### 2.7. Indirect Immunoassay (ELISA) for the Determination of Serum Antibodies IgG1 and IgG2 against Periodontal Pathogens

A blood sample determined serum antibodies IgG1 and IgG2 against 3 selective periodontal pathogens. ELISA was performed as follows. A 96-well plate was covered with 50 μL at a concentration 10 µg/mL of sonicated *P. gingivalis* (ATCC 33277), *T. forsythia* (ATCC 43037), or *A. actinomycetemcomitans* (ATCC 29523) in carbonate buffer (pH 9.6) and incubated overnight. The plates were blocked for an hour at 37 °C with 150 μL of a solution of phosphate buffer (PBS, pH: 7.2) with 1% bovine serum albumin, 1% milk, and avidin. The sera were diluted 1/100 in PBS-Tween-BSA (1%)-biotin solution and incubated at 37 °C per 1 h. Anti-IgG1- dilution 1:5000^ǁǁ^ or anti-IgG2 dilution 1:10,000 in PBS-Tween-BSA (1%) solution were incubated for 1 h at 37 °C. Streptavidin peroxidase in a dilution 1/1500 dilution was incubated for 1 h at room temperature. Three washes were carried out between each incubation period with 250 µL PBS-tween (0.05%) solution for 2 min under stirring. Finally, 50 µL phosphate/citrate buffer solution (0.5 M, pH = 5) was added with O-Phenylenediamine and a concentration of 1 mg/mL was added and activated with H_2_O_2_ for 5 min at room temperature.

The reaction was stopped with a solution of sulfuric acid (2.5 M). Absorbance values were read at 490 nm. The concentration of IgG1 and IgG2 in the samples for each of the microorganisms was measured in triplicate and calculated by linear regression analysis with a known concentration curve (5 µg/mL–0.152 µg/mL, dilution 1:2) of human immunoglobulin IgG1 or IgG2. The reliability of the test was 98% (R^2^ > 98%) with a variation coefficient of <20%.

### 2.8. Statistical Analyses

Descriptive analyses of frequency distribution were carried out for the categorical variables: region (Central—Medellin, Bogotá, and Pacific—Cali), sex (female, male), age (</>55 years), socio-economic status (low, medium, high), according to the classification used by Department of National Planning of Colombia [25], smoking history (never, current, former), current smoking (serum cotinine ≥ 10 ng/mL/<10 ng/mL), alcohol consumption (yes, no), class of drinks (none, soft, strong), blood sugar levels (≥110 mg/dL, <110 mg/dL), and obesity (BMI > 30). Mean, median, and standard deviation were obtained for continuous variables. Antibody levels were categorized into terciles: low, medium, and high. Bivariate analysis was carried out with chi-squared and Fisher’s exact tests. All variables which showed a significance level < 0.10 in the bivariate analysis were included in a logistic regression analysis to establish the odds ratio (OR) and confidence intervals of 95%. Subgroup analyses were conducted by multivariate unconditional logistic regression using a stepwise function by primary outcomes such as CAL (mild vs. moderate and mild vs. advanced CAL), lifestyle (smoking and type of consumed alcohol), genetic factors (SNPs of IL-1a), microbial factors (presence of *P. gingivalis*, *A. actimomycetemcomitans*, *T denticola*, *T. forsythia*, and plaque index), and IgG antibodies (IgG1 and IgG2 subclass against *P. gingivalis*, *T. forsythia*, and *A. actimomycetemcomitans*). The factors associated with subgroup analysis were included in a final model, and obesity as a category was included.

Potential confounders were defined a priori (region and age). For all logistic models, the goodness-of-fit tests for logistic regression (Hosmer–Lemeshow test) were performed. The adjusted and unadjusted models were compared using the likelihood ratio chi-square (G2), Akaike’s Information Criterion (AIC), and the Bayesian Information Criterion (BIC).

A final model was performed with the factors associated with the logistic regression using a generalized ordered logistical regression analysis to determine the factors that kept the proportionality of the association in two levels according to the extent of CAL [26]. All data analysis was undertaken using STATA version 11.

## 3. Results

Table 1 shows the characteristics of the studied population. Medellin presented the highest number of patients with advanced CAL, compared to the other cities (*p* < 0.05). There was a difference in gender among the geographic region, although most of the patients were women. The age was similar for moderate and advanced CAL groups, but the mild CAL group was younger (*p* < 0.05). The periodontal status for the study population is also described in Table 1. The number of remaining teeth between the study groups decreases with the advance of the severity of CAL (*p* < 0.05), and periodontal clinical parameters increase with CAL severity (*p* < 0.05).

Table 2 shows the descriptive and bivariate analysis of lifestyle, systemic, immunogenic, and microbiologic factors, and serum antibodies by CAL. Smoking history, current smoking, and microbial factors such as elevated plaque index, *P. gingivalis*, and *T. forsythia* showed a high association with advanced CAL (*p* < 0.0001). BMI, SNP IL1a, and SNP IL1b, *A. actinomycetemcomitans*, IgG1, and IgG2 antibodies against *P. gingivalis* and *A. actinomycetemcomitans* also were associated with advanced CAL (*p* < 0.05). IgG1 and IgG2 antibodies against *T. forsythia*, alcohol consumption, stress, and glycemia > 120 mg/dL showed no associations in bivariate analysis.

The multivariate logistic analysis for the risk factors associated with CAL severity is shown in Table 3. In both models, the unadjusted models were compared with adjusted models, and the likelihood ratio and BIC provided support for selecting the unadjusted model. In model 1, an association between moderate CAL compared with mild CAL was performed. The socio-demographic factors were not similar among the groups. The region and age (>55 years) were associated with moderate CAL. Microbial factors such as the presence of *P. gingivalis*, *T. forsythia*, and elevated plaque index were associated with moderate CAL. However, IgG1 against *P. gingivalis* antibodies proved to be a protective factor for moderate CAL (OR 0.71, CI 95% 0.55–0.91). Although SNP IL1a and SNP ILb were not associated in the regression model with moderate CAL, obesity was associated (OR 1.40, CI 95% 1.01–1.94). In model 2, an association between advanced CAL and mild CAL was also found. In model 2, advanced CAL was also associated with high levels of IgG2 antibodies against *A. actinomycetemcomitans* (OR 1.63, CI 95% 1.19–2.21). Other factors, such as current smoking (OR 1.97, CI 95% 1.07–3.75) and *P. gingivalis* (OR 3.22, CI 95% 1.89–6.46), were associated with advanced lesions. Nevertheless, *T. forsythia* and IgG1 *P. gingivalis* were associated with the most severe CAL. In this model, plaque index was the most important factor associated with severe CAL (OR 4.59, CI 95% 3.28–6.43). Region and age were also associated with advanced CAL. Obesity was associated with moderate CAL but was not associated with advanced CAL.

A generalized ordered regression model was carried out to determine the factors associated with the extent of CAL. This analysis has been considered in particular due to the outcome variable CAL, which is truly ordered and is composed of three categories; it satisfies the assumption of proportional odds, namely, different models are needed to describe the relationship between, on the one hand, mild versus moderate and severe categories, and on the other hand, mild and moderate versus severe categories. In consequence, this will cause the model to be more parsimonious (Table 4).

For logistic regression, model 1 included all variables associated with unconditional logistic regression models, and model 2 included only factors that showed proportionally in OR in the two levels. Model 1 proved to be the most adjusted model. The factors that followed the assumption of proportionality for both levels that showed the same value of OR were region, age, smoking, plaque index level, *P. gingivalis*, *T. forsythia*, and IgG2 antibodies against *A. actinomycetemcomitans*; therefore, the severity of CAL can be attributed to these factors. Obesity and IgG1 antibodies against *P. gingivalis* were only associated with one level and did not follow the course of proportionality; hence, these cannot be associated with the extent of severity of CAL.

## 4. Discussion

Periodontal disease has been considered a multifactorial disease associated with immunogenic, microbial, and unhealthy lifestyle habits like irregular dental plaque control, excessive alcohol consumption, and smoking. In this study, bacterial factors were linked to the severity of CAL. High supragingival plaque level was relevant for moderate and advanced CAL and an important factor for the severity of CAL. The best available evidence indicates that supragingival prophylaxis and subgingival debridement are comparable concerning the probing depth and clinical attachment level outcomes after 12 months of non-surgical treatment [27]. However, in long-term studies, only supragingival plaque control fails to prevent further periodontal tissue destruction in subjects with advanced periodontal disease [28,29]. In patients with periodontitis, subgingival debridement in conjunction with supragingival plaque control is more effective for reducing probing pocket depth and improving the clinical attachment level than supragingival plaque control alone [30]. Likewise, strict plaque control performed during and after periodontal treatment improves probing depth and clinical attachment level and reduction in the proportions of red and orange complexes in periodontitis [31]. In fact, the supragingival plaque composition seems to be like the subgingival plaque but with a lower proportion of periodontal pathogens, and the supragingival biofilm appears to be a reservoir of periodontopathic microorganisms for the colonization of subgingival plaque [32]; hence, high levels of supragingival plaque can continuously increase the risk of clinical attachment loss. It is possible that high levels of supragingival plaque for long periods might increase the risk of clinical attachment loss by plaque maturation. It augments the inflammatory response in gingival crevicular fluid as the bacterial biomass of the human microbiome escalates alongside increasing periodontal inflammation [33]. Nevertheless, these hypotheses require validation in a clinical context.

In this study, *P. gingivalis* and *T. forsythia* were associated with advanced CAL. The evidence supports the association of these microorganisms with the progression of periodontal disease [34,35,36,37]. Nevertheless, individuals harboring microorganisms in subgingival plaque generally respond with a humoral immune response against them [38]. Plasma IgG levels against these pathogens are highly stable for 15 years in subjects with and without periodontitis and can be a good marker of infection history [39]. In this study, it was only possible to establish an association between advanced CAL and serum markers for *A. actinomicetemcomitans*. High antibodies against *A. actinomycetemcomitans* reflect periods of active disease and recurrence in periodontitis [40,41]. Its detection is a significant risk factor for the onset of attachment loss and disease progression [42]. However, the presence of *A. actinomycetemcomitans* was not associated with the severity of CAL. It is possible that this is due to the observed low frequency of this microorganism in the subgingival plaque. Nevertheless, its levels were lower in mild CAL compared with moderate and advanced CAL. The presence of IgG2 antibodies against *A. actinomycetemcomitans* supports the hypothesis that the increase in severity of CAL is associated with the history of *A. actinomycetemcomitans* infection.

There is strong evidence of the association between smoking and the increase in the severity of CAL in this study. Current smoking significantly predicts clinical attachment loss in longitudinal studies [42,43]. A negative effect of smoking on bone regeneration and the response to periodontal treatment is known from a systematic review and meta-analysis, confirming the importance of this factor [6]. Otherwise, alcohol consumption frequency was not associated with this population’s prevalence or severity of periodontitis. However, although the relationship between periodontitis and alcohol use is still controversial [44], other populations have found an association between periodontitis and a high frequency of alcohol consumption [45,46].

Here, several putative factors for periodontitis were studied. A meta-analysis that evaluated associations of genetic polymorphisms and periodontal disease revealed a greater frequency of IL-1a and IL-b polymorphisms for developing periodontitis; however, this association is variable between regions [47]. In this study, SNPs of the IL gene were not associated with periodontal disease severity. Other types of polymorphism need to be studied to determine their influence on periodontitis severity among Latin Americans [48,49].

Obesity was a risk for the presence of periodontitis but not for advanced CAL. A systematic review of longitudinal studies showed evidence of a stronger association of periodontal disease with BMI [15,16]. However, the proof of a poor response to non-surgical periodontal therapy in the obese is still not consistent [49]. This study shows an association between obesity and moderate CAL in a multivariate model. However, due to the lack of association with advanced CAL, a direct relationship between obesity and the severity of CAL in periodontal patients cannot be demonstrated.

In this study, the selection of participants was not at random, and a sequential allocation of patients attending clinics of universities was used. This was not a representative sample of the general population of the regions studied but individuals who attend university service. There was a low prevalence of obesity, which could influence the lack of association with severe forms of periodontitis. IL-1a and IL-1b SNPs were the factors with the most data missing. However, these missing data did not appear to impact the analysis. Moreover, Hardy–Weinberg balance on genotypic frequency analysis did not show differences between cases and controls in this population. Hence, an examination of a randomized, representative sample from the study population could address these limitations. Additional investigations featuring these attributes and larger sample sizes are imperative to elucidate the present findings.

To summarize, in this study, two different analyses were carried out. The logistic regression can estimate the association of the independent variables with CAL compared with the control (mild CAL); nevertheless, this analysis cannot estimate the effect of these variables in the extent of CAL. Finally, CAL is an ordinal variable, and the best analysis for establishing the impact of the factors in its increase is the generalized ordered logistic model.

## 5. Conclusions

This study reaffirms the significance of previously identified risk factors linked to the severity of periodontitis. Specifically, aging, plaque accumulation, and IgG2 to *A. actinomycetemcomitans* antibodies emerged as the most influential factors associated with advanced clinical attachment loss. Conversely, genetic factors, obesity, and stress were found to be inadequate in explaining variations in the extent of CAL.

## Figures and Tables

**Table 1:** Demographic and periodontal clinical characteristics of population.

Parameter	Mild CAL		Moderate CAL		Advanced CAL	
	Total	Percentage	Total	Percentage	Total	Percentage
Subjects	223/667	33.4%	256/667	38.3%	188/667	28.1%
Cities *						
Medellín	51/226	22.6%	88/256	38.9%	87/188	38.5%
Bogotá	126/297	42.2%	100/256	33.6%	71/188	23.9%
Cali	46/144	31.9%	68/256	47.2%	30/188	20.8%
Gender *	F: 163/223 M: 60/223	73.0% 27.0%	F: 166/256 M: 90/256	64.8% 35.2%	F: 120/188 M: 68/188	63.8% 36.2%
Socio-economic Status *	L: 140/223 ME: 83/223	62.7% 37.3%	L: 183/256 ME: 73/256	71.4% 28.6%	L: 152/188 ME: 36/188	80.8% 19.2%
Age (Mean ± SD) *	45.0 ± 7.7 ^b,c^		48.4 ± 9.19 ^a^		48.3 ± 9.8 ^a^	
No. of teeth *	25.8 ± 3.3 ^b,c^		24.1 ± 4.1 ^a,c^		23.1 ± 3.8 ^a,b^	
PD (Mean ± SD) *	2.2 ± 0.4 ^b,c^		2.7 ± 0.5 ^a,c^		3.8 ± 0.9 ^a,b^	
CAL (Mean ± SD) *	1.3 ± 0.6 ^b,c^		2.8 ± 0.6 ^a,c^		4.8 ± 1.1 ^a,b^	
Bleeding (%) *	36 ± 25 ^b,c^		52 ± 28 ^a,c^		67 ± 28 ^a,b^	
Gingival Index (%) *	42 ± 30 ^b,c^		55 ± 31 ^a^		67 ± 33 ^a,b^	

F = female; M = male; L = low; ME = medium; PD: probing depth full-mouth; CAL: clinical attachment loss in full-mouth; IG: gingival index. * ANOVA, Kruskal–Wallis, Mann–Whitney U, or Chi-square test. *p* < 0.05; ^a^ differences with mild CAL; ^b^ differences with moderate CAL; ^c^ differences with advanced CAL.

**Table 2:** Descriptive and bivariate analyses of lifestyle, systemic, immunogenetic, and microbial factors, and serum antibodies against periodontophatogens by CAL.

**Independent Variables**	**Clinical Attachment Loss**								
	**Mild CAL** **n = 223**		**Moderate CAL** **n = 253**		**Advanced CAL** **n = 188**		**Missing Data**		***p*-Value**
	**F**	**%**	**F**	**%**	**F**	**%**	**F**	**%**	
LIFESTYLE									
Smoking History									
Negative	168	76	179	70.7	105	55.8	5	0.7	<0.0001
Current	31	14	44	17.4	55	29.3			
Former	22	10	39	11.9	28	14.9			
Current Smoking									
Negative	190	85.2	209	82.6	133	70.7	5	0.9	<0.0001
Positive	31	14.8	44	17.4	55	29.3			
Alcohol consumption									
Not	195	88.2	220	88.3	168	89.1	9	1.3	NS
Occasional	20	9	21	8.4	15	8.1			
Frequent	6	2.7	8	3.2	5	2.7			
Stress (Cortisol) mean ± SD	13.31 ± 1.20		13.04 ± 0.75		13.49 ± 0.72		12	1.8	NS
SYSTEMIC FACTORS									
BMI									
Normal	132	59.2	131	51.6	104	55.6	3	0.4	<0.05
Overweight	81	36.3	85	33.5	64	34.2			
Obesity	10	4.5	38	14.9	19	10.2			
Glycemia									
<120 mg/dL	191	85.6	204	83.9	157	83.5	10	1.4	NS
>120 mg/dL	32	14.3	39	16	31	16.4			
IMMUNOGENETIC FACTORS									
SNP IL-1b									
C-C	147	66.8	162	66.2	135	73.4	16	2.3	<0.05
T-T	18	8.2	29	11.8	22	12.1			
CT/TC	55	25	44	18	23	12.6			
SNP IL-1a									
CC	127	60.5	125	53.2	124	55.9	16	2.4	<0.05
T-T	21	10	45	19.1	51	23			
CT/TC	58	27.6	57	24.2	43	19.3			
MICROBIAL FACTORS									
Plaque index									
Low	124	56.4	70	28.6	25	13.5	0	0.0	<0.0001
Middle	65	29.6	95	38.7	51	27.6			
High	31	14	80	32.7	109	58.9			
*P. gingivalis*									
Not	115	51.8	80	31.3	45	23.9	2	0.3	<0.0001
Yes	107	48.2	145	68.7	143	76.1			
*T. forsythia*									
Not	119	53.6	68	26.6	52	22.3	2	0.3	<0.0001
Yes	103	46.4	187	73.4	143	77.6			
*T. denticola*									
Not	160	71.3	158	62.9	106	56.3	2	0.3	<0.05
Yes	63	29.7	93	37.1	82	43.7			
*A. actinomycetemcomitans*									
Not	215	96.8	232	90.9	169	89.9	2	0.3	<0.05
Yes	7	3.3	22	9.1	22	10.1			
SERUM ANTIBODIES									
IgG1 *P. gingivalis*									
Low	76	34.1	99	38.7	47	25	0	0.0	<0.10
Middle	61	27.3	92	35.9	69	36.7			
High	86	38.6	75	25.4	62	38.3			
IgG1 *T. forsythia*									
Low	75	33.6	82	32.4	63	33.5	0	0.0	NS
Middle	65	29.1	80	31.6	73	38.8			
High	83	37.3	91	36.0	52	27.7			
IgG1 *A. actinomycetemcomitans*									
Low	99	39.1	80	31.6	56	29.7	0	0.0	<0.10
Middle	84	33.2	81	32.1	64	34			
High	70	27.6	92	36.3	68	36.3			
IgG2 *P. gingivalis*									
Low	91	41	81	31.7	50	26.6	0	0.0	<0.05
Middle	67	30.2	83	32.4	73	38.8			
High	64	28.8	92	35.9	65	34.6			
IgG2 *T. forsythia*									
Low	88	39.4	84	33.2	51	27.1	0	0.0	NS
Middle	66	29.6	87	34.4	68	36.2			
High	69	31.1	82	32.4	69	36.7			
IgG2 *A. actinomycetemcomitans*									
Low	92	41.4	84	32.8	46	24.5	0	0.0	<0.05
Middle	65	29.3	92	35.9	75	34.6			
High	65	29.3	80	31.3	67	40.9			

**Table 3:** Logistic regression models. Risk factors associated with clinical attachment loss.

	**MODEL 1**		
**BASE OUTCOME Mild CAL**		**Unadjusted OR (IC 95%)**	**Adjusted OR (IC 95%)**
**Compared with moderate CAL**			
Base comparison			
CITIES		1	1
Bogotá		1.51 ** (1.14–1.99)	1.85 (0.90–3.80)
Other			
AGE		1	1
<55 years		1.50 ** (1.15–1.95)	2.36 † (1.22–4.55)
>45 years			
MICROORGANISMS		1	1
*P. gingivalis* positive		1.95 ** (1.23–3.08)	2.78 ** (1.07–7.19)
*T. forsythia* positive		1 2.10 ** (1.33–3.32)	1 5.02 ** (1.95–12.9)
ANTIBODIES	low	1	1
IgG1 *P. gingivalis* Middle-High		0.71 ** (0.55–0.91)	0.70 (0.42–1.17)
PLAQUE INDEX	low	1 2.25 † (1.72–2.95)	1 1.91 (1.32–3.25)
Obesity		1	1
Positive		1.40 ** (1.01–1.94)	1.41 (0.69–2.84)
	**MODEL 2**		
**BASE OUTCOME Middle CAL**		**Unadjusted OR (IC 95%)**	**Adjusted OR (IC 95%)**
Compared with advanced CAL			
Base comparison			
CITIES		1	1
Bogotá		1.85 † (1.31–2.61)	1.32 (0.50–3.48)
Other			
AGE			
<45 years		1	1
>55 years		1.63 ** (1.19–2.23)	2.70 ** (1.12–6.54)
MICROORGANISMS		1	1
*P. gingivalis* positive		3.22 † (1.89–6.46)	2.54 (0.88–7.31)
ANTIBODIES	low level	1	1
IgG2 *A. actinomycetemcomitans* Middle-High Level		1.63 ** (1.19–2.21)	1.98 ** (1.06–3.69)
PLAQUE INDEX Middle-High level	low	1 4.59 † (3.28–6.43)	1 5.65 † (2.97–10.7)
SMOKING	never	1	1
Current		1.97 ** (1.07–3.65)	1.76 (0.49–6.27)

Middle CAL (clinical attachment loss) < 2 mm, MP = moderate CAL > 2 < 3.5 mm, advanced CAL ≥ 3.5 mm. ** *p* < 0.05. † *p* < 0.000. Model 1: comparison of unadjusted OR and adjusted OR (adjusted to age and region). Likelihood ratio *p* = 0.36. Difference in BIC = unadjusted 597.7816 − adjusted 658.5311 = −60.7495 provides positive support for the unadjusted OR. Model 2: comparison of unadjusted OR and adjusted OR (adjusted to age and region). Likelihood ratio *p* = 0.10. Difference in BIC = unadjusted 433.6645 − adjusted 489.7469 = −446.0824 provides positive support for the unadjusted OR.

**Table 4:** Generalized ordered regression model. Risk factors associated with CAL.

**Level 1**	**MODEL 1**	**MODEL 2**
**BASE OUTCOME Mild CAL**	**OR (IC 95%)**	**OR (IC 95%)**
**Compared with Moderate + Advanced CAL**		
CITIES Bogotá	1	1
Other	1.34 ** (1.09–1.64)	1.58 † (1.25–2.01)
AGE < 45 years	1	1
>55 years	1.46 ** (1.21–1.77)	1.45 † (1.20–1.75)
MICROORGANISMS		
*P. gingivalis* Negative	1	1
Positive	1.93 ** (1.35–2.76)	2.44 † (1.77–3.38)
*T. forsythia* Negative	1	
Positive	1.88 ** (1.30–2.70)	
ANTIBODIES		
Low levels	1	
IgG1 *P. gingivalis*	0.85 (0.67–1.06)	
Middle-High		
Low levels	1	
IgG2. *A. actinomycetemcomitans*	1.50 † (1.23–1.81)	1.48 † (1.22–1.78)
Middle-High		
PLAQUE INDEX Low	1	1
Middle-High	2.69 † (2.20–3.29)	2.76 † (2.27–3.36)
OBESITY Negative	1	
Positive	2.99 ** (1.40–6.36)	
SMOKING Negative	1	1
Positive	1.94 ** (1.31–2.87)	1.88 ** (1.28–2.76)
**Level 2**		
**BASE OUTCOME**	**Mild + Moderate CAL**	
**Comparison with advanced CAL**		
REGION	1	1
Bogota	1.34 ** (1.09–1.64)	1.54 † (1.21–1.96)
Other regions		
AGE < 45 years	1	1
>55 years	1.46 † (1.21–1.77)	1.46 † (1.21–1.76)
MICROORGANISMS		
*P. gingivalis* Negative	1	1
Positive	1.93 † (1.35–2.76)	1.89 † (1.33–2.69)
*T. forsythia* Negative	1	
Positive	1.88 ** (1.30–2.70)	2.33 † (1.56–3.49
ANTIBODIES Low level	1	
IgG1 *P. gingivalis*	1.42 ** (1.12–1.80)	
Middle-High		
Low levels	1	1
IgG2 *A. actinomycetemcomitans* Middle-High Level	1.50 † (1.23–1.81)	1.50 † (1.24–1.81)
PLAQUE INDEX Low level	1	1
Middle-High level	2.69 † (2.20–3.29)	2.67 † (2.19–3.26)
OBESITY Negative	1	
Positive	0.94 (0.50–1.74)	
SMOKING Never	1	1
Current	1.94 ** (1.31–2.87)	1.86 ** (1.26–2.74)

Middle CAL (clinical attachment loss) < 2 mm, MP= moderate CAL > 2 < 3.5 mm, advanced CAL ≥ 3.5 mm. ** *p* < 0.05. † *p* < 0.000. Level 1: comparison of mild vs. moderate and advanced CAL. Level 2: comparison mild and moderate vs. advanced CAL. Difference in BIC: model 1 = 1276.918, model 2 = 1287.819 = −10.901; this provides positive support for model 1.

## Data Availability

Data are unavailable due to privacy or ethical restrictions.
